# The Laws of Cholera

**Published:** 1866-06

**Authors:** 


／# HALL'S JOURNAL OF HEALTH.

Our Legitimate Scope is almost boundless: for whatever begets pleasurable and harmless feelings, promotes Health; and whatever induces disagreeable sensations, engenders Disease.

WE AIM TO SHOW HOW DISEASE MAY BE AVOIDED, AND THAT IT IS BEST, WHEN SICKNESS COMES, TO TAKE NO MEDICINE WITHOUT CONSULTING A PHYSICIAN.

Vol. XIII.] JUNE, 1866. [No. 6.

### THE LAWS OF CHOLERA.

The radical cause of Asiatic Epidemic Cholera is a something added to the atmosphere which does not materially belong to it. That radical, originating cause cannot, of itself, bring on an attack of cholera any more than powder will explode without the application of a spark. This spark, as to cholera, is any thing which debilitates the human body; which depresses its vital power, the general health, below its natural standard. Cholera cannot attack a man when he is in good general health. No one single case of the kind has ever yet been brought to light in any part of the world, after a careful medical and anatomical investigation by competent professional men. In every single case where the person attacked was reported by friends and neighbors to have been in perfect health up to the moment of attack, it has never failed to have been found, when calmly investigated, that some organ of the body had been out of order; some function of the system had been suspended; some unusual and unhealthful condition of the organism had been present, or some surroundings of the patient had been changed, so as to invite disease; that is, some agent had been at work which was calculated to undermine the physical, moral or mental health of the patient, and thus debilitate the general system.

FEAR.—Sudden emotions of alarm or apprehension make some persons "as weak as water." Surgeon Phillips of the United States Army, relates in the November number of the Medical and Surgical Reporter, of Philadelphia, that a sentry who had to do duty in the passage of a cholera ward, was at his post quite well, but became alarmed as soon as he learned that there were cases of cholera in the rooms; although he could not see them he became very nervous, no assurance could quiet his apprehensions, so that he had to be relieved, and "died within two hours." It is, therefore, clear that fear so debilitates the body as to make it susceptible of an attack of malignant cholera in a cholera atmosphere. Physicians even of moderate experience, know that in the healthiest times, and among persons in good general health, diarrhœa is an immediate result of any depressing excitement, and Asiatic cholera is only an exaggerated diarrhœa.

LIQUOR of any kind makes a man stronger for the time being, but when it begins to die out, he is weaker than he was before he drank anything, and in that condition he is a fit subject for an attack of cholera when the disease is prevalent. A writer in the Richmond Medical Journal for February, 1866, states from personal knowledge that when the cholera appeared in the lower part of Wheeling, a few cases here and there, every case died; every victim had been in the habit of "taking a drink."

EATING HEARTILY.—A man is not as able to work, nor a horse to travel immediately after a full meal as he was before, or as he would be an hour or two later: there is a want of vigor, or animation, of strength; hence, hearty feeding in cholera times invites the disease.

Cleanliness.—One fact has been observed all over the world. In all localities where fevers ordinarily prevail cholera feeds and becomes more unmanageable and malignant. All know that fevers most abound in warm weather; in marshy places; in flat, low, wet, and filthy localities. New Orleans is a perfect type of such a situation. Taking four years preceding the war ;and fours years during the war fewer persons died of yellow fever in New Orleans, in all these four years, than died in a single year previously, simply because the United States authorities compelled universal cleanliness. The streets and yards of dwellings were kept clean and dry by judicious draining and scrubbing; and cleansing and whitewashing were the order of the day.

The city of Worcester, England, has been twice ravaged by the cholera; to prevent a third visit, the authorities inaugurated such a system of "cleansing" in every street and alley and dwelling that "not a house was entered" by the destroyer, while the most frightful desolation prevailed among the neighboring cities.

In the "Metropolitan Buildings," the great tenant house of London, in which "the health regulations were complete," and which contained five hundred occupants, there was not a single case of cholera; and yet, in the same district, the epidemic was very fatal. In one of our own large cities one ward was thoroughly inspected and cleaned, in anticipation of the advent of cholera; it came, and only one house suffered. On a more minute inspection, a heap of noisome house offal of several years accumulation was found in a dark corner of the cellar, and which had been overlooked.

The cholera fell fatally on a village fifty miles from Montreal, with which there was daily intercourse; the disease did not spread around that village, not a single case occurred in all that long highway af trade and travel; but after a time it did appear in Montreal itself.

Between Wheeling, Va., and Bridgeport, a distance of a half-a-mile, a ferry plies, an island and two branches of the river intervened; the cholera ravaged Wheeling five weeks, and then appeared for the first time and suddenly at Bridgeport, destroying nearly the entire population.

Two emigrant vessels left Havre in October; 1848, one for New Orleans, the other for New York; Havre was "unaffected" at the time of their departure. Sixteen days out, the cholera appeared on board the New York vessel; and 37 days out on the New Orleans ship; the disease did not spread at New York, but it did in New Orleans. Several vessels and steamships have left affected European ports and arrived at our shores without a single case of the disease. The cholera first nestled several years about the mouths of the Ganges and then took a general north-westerly course which, in the main, it continued until it encircled the globe.

In 1831, the cholera appeared in Berlin, spread north to the Baltic, thence west a thousand miles to London, thence south, over two hundred miles, to Paris at the end of six months. But Paris is some five hundred miles only from Berlin, between which two places there was perhaps the most constant inland communication by travel and traffic in all Europe.

In May, 1865, the Cholera appeared at Cairo, in June at Constantinople, near a thousand miles North; thence west, over a thousand miles to Marseilles, and in five months, it appeared in Paris, over four hundred miles north, and yet there is an incessant stream of travel by land between these two places. In one month, it traveled a thousand miles from Cairo to Constantinople, and yet was five months in traveling from Marseilles to Paris, one-half the distance; these cases show that great lines of travel and trade do not always carry the Cholera along, and as natures laws are always uniform, under the same circumstances, some other theory for Cholera propagation is needed, one which will answer all the conditions; meanwhile we cannot do better than to fall back on the hypothesis already taken in this article, that two things are always essential to the presence of epidemic Cholera in any place, first, there must be a cholera atmosphere, and it must meet acting and generally prevalent causes of bodily or mental debility, which are fear and despondency as to the mind; and as to body, the exciting cause of epidemic fevers, which is miasm, an emanation from the earth, wherever there is heat of eighty degrees, and moisture and vegetable decay, such as leaves, wood, grass, &c., in bottom or flat and made lands, and the great practical lesson is, that should the cholera appear in the United States during this present year of 1866, the fearful, the infirm, the debilitated should, as far as possible, and as soon as it can be done, remove to high land situations, and remain there until there have been several frosts at their own homes.

As to all these facts, two things we do not as yet know. First, we do not know what that is, which added to an atmosphere usually healthful makes it a cholera atmosphere. Second, we do not know the law of the spread of a cholera atmosphere. But we do arrive at certain practical conclusions, which are of immense sanitary and commercial importance.

First. A cholera atmosphere is not necessarily diffused by means of lines of travel and trade. Second. Cholera cannot be quarantined from our shores. Third. The fundamental cause of epidemic Asiatic Cholera, is a cholera atmosphere. Fourth. The immediate exciting cause of this disease is filth, or a debilitated condition of the system. Fifth. There can be no epidemic Asiatic Cholera unless the cholera atmosphere and the exciting causes are both present at the same time. Sixth: The ravages of the disease in any community is measured by the degree of the prevalence of the exciting causes ; where dampness, warmth and filth most prevail, there will the scourge be proportionably malignant.

The facts in this article seem to authorize the conclusion that a cholera atmosphere spreads by an unknown law ; that as it does not always advance in the track of wind and tide and travel and traffic, those cases which are given as proofs of this are mere coincidences. Nature's laws are infallible in their action ; under the same circumstances they act in the same way ; and as, in some of the statements made, it did not go in the direction of wind and travel, inter-communication cannot be a law of the spread of cholera. Quarantine looses millions of money, and results in incalculable discomfort and inconvenience. Instead of incurring these, when no one claims they can be always efficient, it would be wiser to adopt measures which all admit will ward off the disease, as facts given clearly show ; measures which do not cost a tithe as much as a quarantine and which cannot but result in an incalculable amount of public good. The dictate of a true philanthropy and of undoubted wisdom is to direct attention to the securement of as perfect cleanliness as possible, in person, in clothing, in habitation,. cellar, attic, street, alley, neighborhood. As the removal of filth, and cleanliness, and securing dryness by draining, have in so many cases been followed by an entire exemption from cholera, and as embargo and quarantine are at most doubtful, and in some cases have been clearly unavailing, it seems to be the dictate of a sound common sense to direct attention to the certain instead of the uncertain.

In view of the above statements, a theory seems to present itself, which will meet all the facts detailed above. That a cholera atmosphere causes cholera only when it meets with filth or any of the causes of bodily debilitation; and as all human means have failed to arrest the spread of a cholera atmosphere, the removal of the causes of, or the condition of, bodily debilitation, is our only hope for the prevention of the disease in any specified locality; and as such a removal is a specific, and is everywhere practicable, it is our own fault if we suffer from the scourge.

The ships which left an "uninfected" port had the immediate cause of cholera aboard; want of cleanliness and vigorous health among a crowd of steerage emigrants, and meeting with a cholera current in their passage across the ocean, as the track of the gulf stream, or of a tornado is met, the disease manifested itself; one ship found the cholera atmosphere in New Orleans and cholera material too, there it spread; the writer was a resident of that city at the time; the other ship found no cholera atmosphere in New York, and the scourge did not show itself.

The ships which left infected ports, and crossed over without a case, were clean vessels, had few passengers, who felt the necessity of attendance to the laws of health, and escaped a visitation; all going to show that epidemic Asiatic Cholera can only occur where a cholera atmosphere, the primary cause, meets with the immediately exciting cause, which is the want of cleanliness in person, habitation and neighborhood. Single occasional cases, called sporadic, are not taken into account; the aim has been to establish great general principles, and the attention of scientific men and those of leisure and cultivation is invited to a collection of well authenticated whole facts, and if they all are explainable on the sentiments we have advanced, then a great advance is made, if they cannot be explained they may lead eventually to the truth.

